# Biodegradation of Halloysite Nanotubes-Polyester Nanocomposites Exposed to Short Term Seawater Immersion

**DOI:** 10.3390/polym9080314

**Published:** 2017-07-28

**Authors:** Mohd Shahneel Saharudin, Jiacheng Wei, Islam Shyha, Fawad Inam

**Affiliations:** 1 Department of Mechanical and Construction Engineering, Faculty of Engineering and Environment, Northumbria University, Newcastle upon Tyne NE1 8ST, UK; jiacheng.wei@northumbria.ac.uk (J.W.); islam.shyha@northumbria.ac.uk (I.S.); fawad.inam@northumbria.ac.uk (F.I.); 2 Institute of Product Design and Manufacturing (UniKL IPROM), Universiti Kuala Lumpur, Cheras, 56100 Kuala Lumpur, Malaysia

**Keywords:** biodegradation, polyester-nanocomposites, halloysite nanotubes, seawater

## Abstract

Halloysite nanotubes (HNTs)-polyester nanocomposites with four different concentrations were produced using solution casting technique and the biodegradation effect of short-term seawater exposure (120 h) was studied. Monolithic polyester was observed to have the highest seawater absorption with 1.37%. At 0.3 wt % HNTs reinforcement, the seawater absorption dropped significantly to the lowest value of 0.77% due to increase of liquid diffusion path. For samples tested in dry conditions, the *T*_g_, storage modulus, tensile properties and flexural properties were improved. The highest improvement of *T*_g_ was from 79.3 to 82.4 °C (increase 3.1 °C) in the case of 0.3 wt % HNTs. This can be associated with the exfoliated HNTs particles, which restrict the mobility of polymer chains and thus raised the *T*_g_. After seawater exposure, the *T*_g_, storage modulus, tensile properties and flexural properties of polyester and its nanocomposites were decreased. The Young’s modulus of 0.3 wt % HNTs-polyester dropped 20% while monolithic polyester dropped up to 24% compared to their values in dry condition. Apart from that, 29% flexural modulus reduction was observed, which was 18% higher than monolithic polyester. In contrast, fracture toughness and surface roughness increased due to plasticization effect. The presence of various microbial communities caused gradual biodegradation on the microstructure of the polyester matrix as also evidently shown by SEM images.

## 1. Introduction

Unsaturated polyesters have been widely used in many applications such as marine pipes, coatings and storage tanks for oil and gas industry [1,2,3,4,5] because of their cheaper price and chemical resistant property. Unsaturated polyesters are also used as matrix materials, predominantly with nano-particles reinforcement since they have good high dimensional stability and low moisture absorption [6].

Unsaturated polyesters polymerize at relatively higher temperatures and tend to have higher cure shrinkage than epoxies. Apart from that, the tensile strength and Young’s modulus of polyester are lower than those of epoxy resins [7]. For industrial applications, polymers such as epoxy and polyester are also vulnerable to mechanical properties degradation when exposed to marine environment [5,8]. Many fillers such as clay, layered silicates, alumina, carbon fiber, graphene and CNTs have been used until today to improve mechanical properties of polymers [9,10].

It is noted from literature that clay/polymer nanocomposites offer incredible enhancement in many engineering applications for polymers with low filler content [11,12,13]. Clay based particles can be found in many applications such as civil engineering structures and coatings as they can increase service life of materials exposed to aggressive environments [14]. Clay based particles such as montmorillonite, bentonite and halloysite have gained great attention in recent years. Unlike montmorillonite and bentonite, halloysite is less researched material compared to other clay-based particles. Only in recent years, halloysite is regarded as one of most promising natural nanoscale materials [15]. It has been reported that approximately 30,000 tons of halloysite clay minerals are excavated worldwide and processed to dispersed nanotubes [16]. The unique crystal structure of halloysite is similar to CNTs, making them a potential candidate to replace expensive materials such as CNTs since they have a tubular structure in nano-size.

Halloysite nanotubes (HNTs) are nontoxic in nature and have wide range of applications for cancer treatment, drug delivery and environment protection [17]. Most recently, Buvhana and Prabakaran reported that the addition of HNTs significantly increased storage modulus of polyamide up to 36% [18]. In addition, an improvement of Young’s modulus up to 26% was reported by Gabr et al. [19]. Most studies seem to agree that the incorporation of HNTs into epoxy will increase its mechanical properties. In view of the above-mentioned research advances, there are great prospects for HNTs based materials since they are also becoming the subject of intense in global research.

It is noted that the dispersion of HNTs in polymer matrix can be enhanced through numerous techniques such as high speed stirrer, high shear mixer and, more commonly, ultrasonication [20]. Previous research studies suggested that sonication was only possible for up to 3% clay loading due to the lower viscosity of the mixture [21]. The dispersion of HNTs in the polyester resin is improved using sonication as it reduces air bubbles at the interface between clay particles and resin molecules. Dispersion also achieved exfoliation of particles. However, the added curing agent caused the viscosity to increase rapidly. In this research, ultrasonication technique was applied to disperse HNTs in polyester matrix. Table 1 presents impact strength, Young’s modulus and flexural modulus of clay-polymers composites. The maximum impact strength was recorded for HNTs-epoxy with 300% improvement compared to monolithic epoxy [21]. Carli et al. reported 63% improvement in Young’s modulus for HNTs-PHBV (synthesized polyesters) [22]. Maximum flexural modulus up to 224% for MMT-epoxy was also revealed by Pavlidou et al. [23]. In summary, these publications suggest that the addition of clay-based particles can significantly improve mechanical properties of its composites.

**Figure 1:**
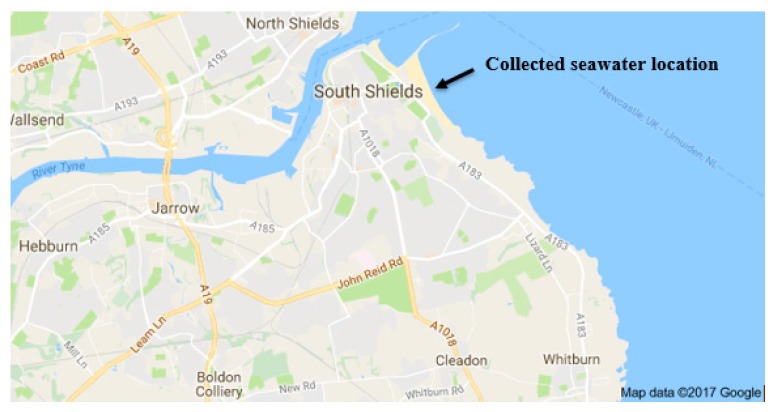
Location of the collected seawater, South Shields Beach, United Kingdom.

The objective of this research was to study the biodegradation of HNTs-polyester nanocomposites exposed to short-term seawater immersion. There is little published data on the biodegradation effect on HNTs-polyester nanocomposites. Most studies only focused on degradation of polymeric materials in marine environment [24,25,26,27,28]. Some durability data with seawater immersion for short-term of immersion must be obtained to predict the durability of HNTs-polyester nanocomposites when exposed in seawater. In this study, the seawater was collected from South Shield Beach and the location in the map is shown in Figure 1.

## 2. Materials and Methods

**Figure 2:**
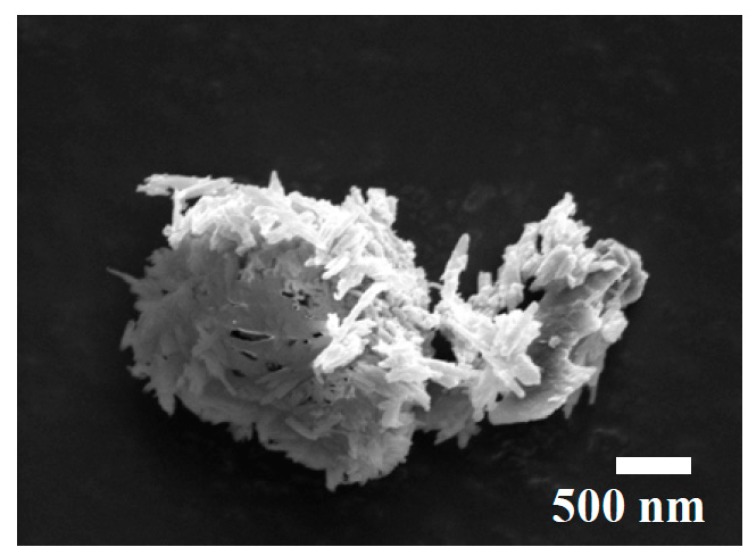
Image of halloysite nanotubes (HNTs).

The polyester resin (NORSODYNE O 12335 AL) and catalyst were purchased from East Coast Fibreglass, Newcastle, UK. The polyester resin has a density of 1.12 g/cm^3^. The catalyst used in this research was methyl ethyl ketone peroxide solution in dimethyl phthalate with density of 1.18 g/cm^3^. The polyester resin and the catalyst ratio was 98:2. The HNTs used in this research were acquired from Sigma-Aldrich, Irvive, UK. Figure 2 shows the SEM image of HNTs. HNTs samples were weighed in Sartorius MC210S (Surrey, UK) machine by analytical balance (with the readability of 0.001 mg) and dispersed in polyester resin by hand mixing for 20 s gently and then sonicated for 30 min through a bath sonicator (Grant MXB6) for uniform dispersion. The duration of sonication was selected based on previous research by Vahedi et al. [39]. The bath sonicator was rated for an average working power output of 89 W. After sonication process, the resin, HNTs and catalyst mixture were poured onto silicon molds and cured for 24 h at room temperature. After that, post curing was performed for 2 h at 80 °C in an oven.

### Characterization

Dynamic storage modulus (E′), and loss modulus (E″) of the samples were analyzed using Dynamic Mechanical Analyzer (DMA 8000, Perkin-Elmer, Waltham, MA, USA). The loss factor tanδ was obtained as the ratio (E”/E’). The glass transition temperature (*T*_g_) was taken as the temperature value at the peak of tanδ curves. Rectangular test specimens of dimensions 15 mm × 6 mm × 3 mm were used with a single cantilever clamp. All tests were conducted by temperature sweep method (temperature ramp from 60 to 100 °C at 5 °C min^−1^) at a constant frequency of 1 Hz. The maximum force of DMA was 10 N and applied during all DMA tests. Scanning Electron Microscopy (SEM) analysis using a FEI Quanta 200, was carried out of the fractured surfaces of flexural specimens to evaluate the fracture modes in the samples. The fractured portions were cut from the specimens and a layer of gold was applied using Emscope sputter coater model SC500A.

To measure the seawater absorption, rectangular specimens with dimensions 80 mm × 10 mm × 4 mm were immersed in seawater at room temperature. After 120 h immersion, the seawater was completely wiped up from the specimen surface by using absorbent paper and weighed with 0.001 g accurate weighing balance. The achieved constant weight was taken to obtain the wt % increase of seawater content. Equation (1) was used for the maximum seawater absorption.

(1) $$W_{C}=\left(W_{t}-W_{o}\right)\times (\frac{100}{W_{o}})$$

Light transmittance of halloysite nanotubes-polyester nanocomposites was recorded at fixed wavelength of 400 nm on cured samples. Five specimens were tested for each set of conditions and mean values were then recorded. Cured samples with concentration of 0.1, 0.3, 0.7 and 1 wt % were fractured to observe the dispersion of HNTs.

**Figure 3:**
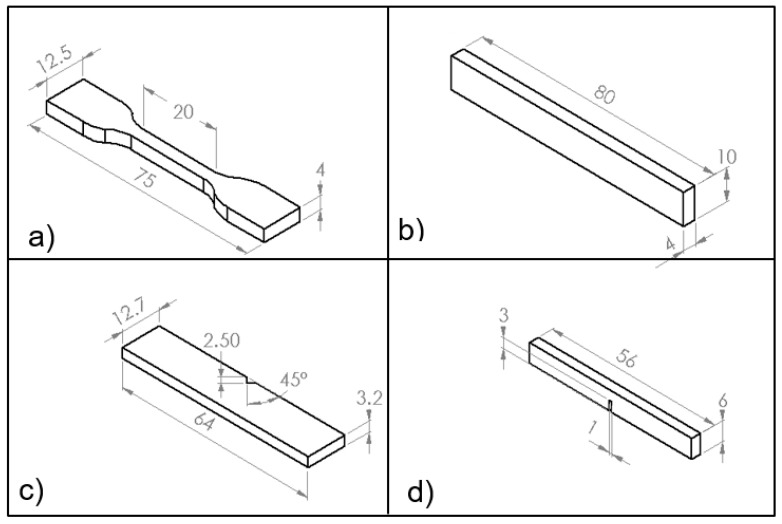
Schematics of samples: (**a**) tensile; (**b**) flexural; (**c**) impact toughness; and (**d**) fracture toughness (All dimensions in mm).

Tensile, three-point bending and fracture toughness tests were performed using Instron Universal Testing Machine (Model 3382). Five samples were tested for each composition and the displacement rate used was 1 mm/min. Tensile properties were carried out according to ISO 527 as shown in Figure 3a with specimen thickness of 3 mm. Three-point bending test was performed according to ISO 178 with dimensions 80 mm × 10 mm × 4 mm. A single edge notch three-point bending (SEN-TPB) was used to investigate mode-I fracture toughness K_1C_ according to ASTM D5045. The dimensions were 3 mm × 6 mm × 36 mm with crack length 3 mm. The notch was made at the mid of sample and tapped to sharpen by a razor blade. The K_1C_ was determined from Equation (2).

(2) $$K_{1C}=\frac{P_{\max}(\frac{a}{w})}{{\mathrm{BW}}^{1/2}}$$

(3) $$f\left(\frac{a}{w}\right)=\frac{\left[\left(2+\frac{a}{w}\right)\{0.0866+4.64\left(\frac{a}{w}\right)-13.32\left(\frac{a}{w}\right)-13.32{\left(\frac{a}{w}\right)}^{2}+14.72{\left(\frac{a}{w}\right)}^{3}-5.6{\left(\frac{a}{w}\right)}^{4}\}\right]}{{\left(1-\frac{a}{w}\right)}^{\frac{3}{2}}}$$

Charpy impact tests were carried out using sample as illustrated in Figure 3c. The impact toughness was calculated using the Equation (4) below [40],

(4) $$\mathrm{Impact}\;\mathrm{toughness}=\frac{mgh\;(\cos\beta -\cos\alpha )}{wt}$$

where *m* is mass of hammer (kg), g is standard gravity (9.8 m/s^2^), *h* is length of hammer arm (m), β is hammer swing up angle of fractured sample (rad), α is hammer lifting angle (rad), *w* is sample width (mm), and *t* is sample thickness (mm).

An Alicona optical microscope was used to study the topographical features of produced samples. The Alicona Infinite Focus optical microscope (G4, Alicona, Raaba/Graz, Austria) was used to generate optical micrographs and measure topographical features. The Alicona optical microscope is a non-contact method (focus-follow method) for topography measurement.

Scanning Electron Microscopy (SEM) analysis, using a FEI Quanta 200, was carried out for the fractured surfaces of tensile specimens to evaluate the fracture modes in the samples. The fractured portions were cut from the specimens and a layer of platinum was applied using Emscope sputter coater model SC500A. The thickness of the coating applied was 5 nm for all samples. In-beam detector was used to observe the fractured surface of the nanocomposites. The detector allows imaging at very short working distances hence excellent resolution can be achieved.

## 3. Results and Discussion

**Figure 4:**
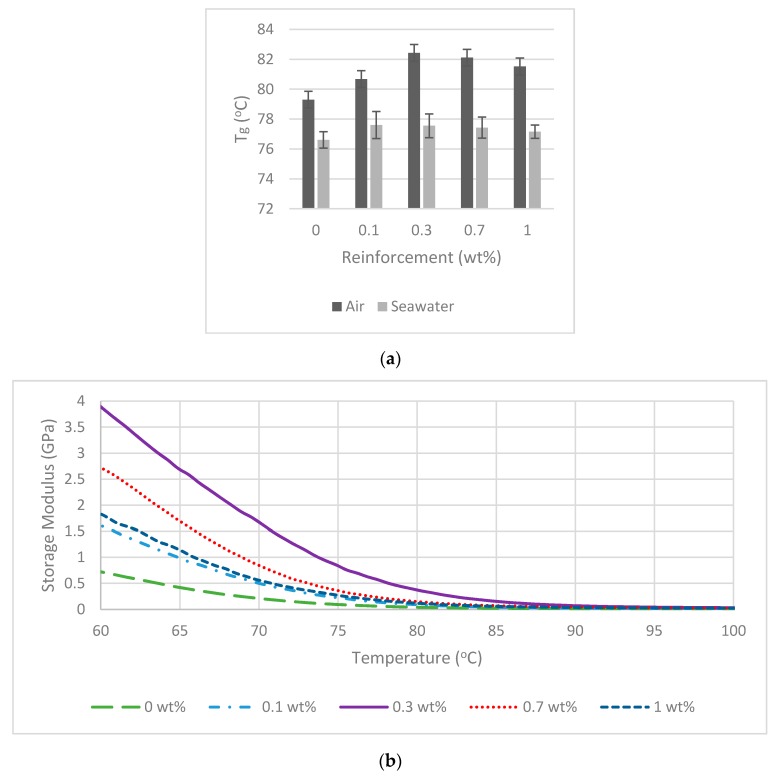

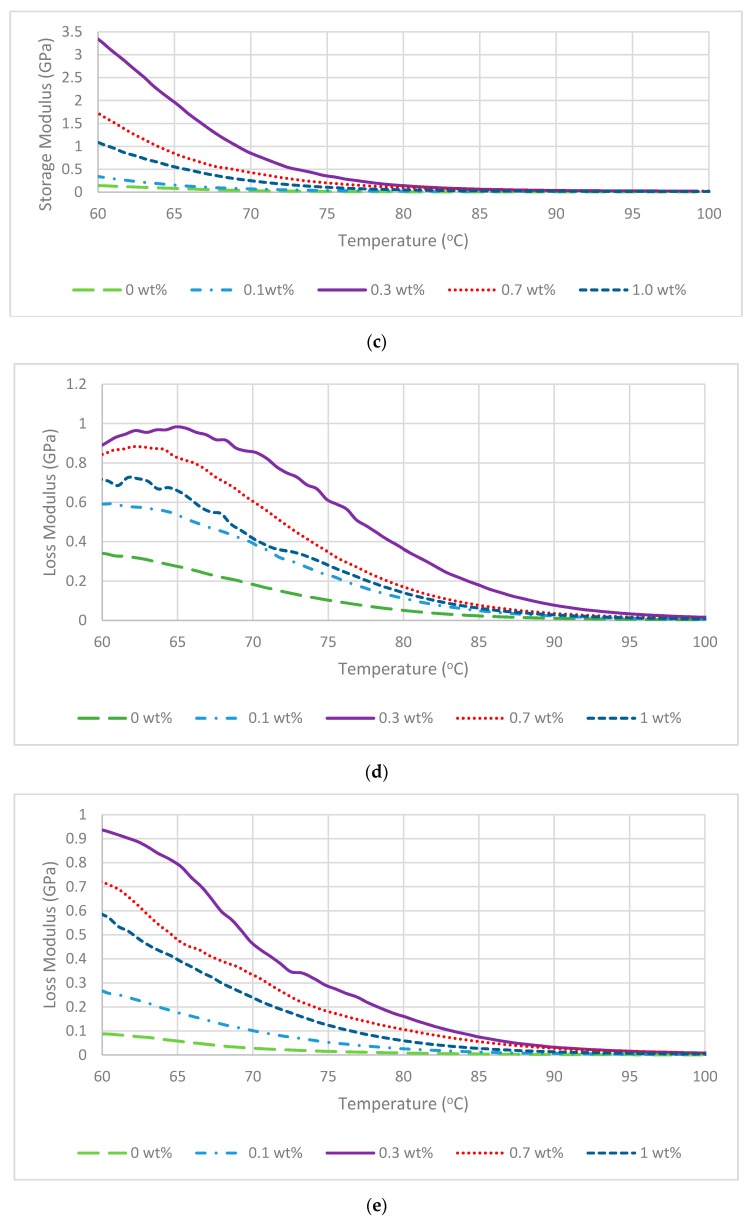
Dynamic mechanical properties: (**a**) glass transition temperature; (**b**) storage modulus of nanocomposites in air; (**c**) storage modulus of nanocomposites after seawater exposure; (**d**) loss modulus of the nanocomposites in air; and (**e**) loss modulus of nanocomposites after seawater exposure.

The variation of *T*_g_ is presented in Figure 4a below. Generally, it has been reported that for nanocomposites systems, *T*_g_ will increase or decrease monotonically with increasing nano filler content. Our results indicate that HNTs increased the *T*_g_ [18]. When the HNTs are evenly dispersed_,_ the high aspect ratio influences the exothermic heat flow temperature by restricting polymer chain mobility that results in *T*_g_ increase. The storage moduli increased while loss moduli decreased for polyester reinforced with HNTs compared to monolithic polyester. In the case of 0.3 wt % HNTs-polyester, the *T*_g_ showed the highest improvement from from 79.3 to 82.4 °C (increase 3.1 °C). This could be linked to the exfoliated HNTS particles which restrict the mobility of polymer chains and thus raised the *T*_g_ [41]. At 0.7 and 1 wt % HNTs reinforcement, the *T*_g_ were 82.1 and 81.5 °C, respectively. The presence of aggregates and poor interaction between HNTs and polymer matrix reduced the *T*_g_ value at higher HNTs concentrations. The storage moduli and loss moduli are also shown in Figure 4. Storage moduli, loss moduli and *T*_g_ were decreased after seawater exposure. The minimum *T*_g_ recorded was monolithic polyester with 76 °C. The maximum *T*_g_ of 77.6 °C was recorded in the case of 0.3 wt % HNTS-polyester nanocomposites. The decrease in *T*_g_ after seawater is the sign of degradation within the samples. Plasticization due to moisture absorption leads to ductile failure in the matrix also cause the same degradation effect [42]. At glass transition temperature, *T*_g_, monolithic polyester recorded the lowest storage modulus (11.8 MPa). Interestingly, the value of storage modulus at 0.3 wt % HNTs reinforcement showed an improvement of 380%. That was the highest value that was achieved in this research. In comparison to monolithic polyester, the HNTs reinforcement in both conditions (air and seawater) was efficient in improving the dynamic mechanical properties. Plasticization and biodegradation may have caused the deterioration in dynamic mechanical properties of the nanocomposites, however they can be reduced by incorporating HNTs. Living organisms such as microbes are also capable to cause chemical change via reducing molecular weight polymer fragments [28].

**Figure 5:**
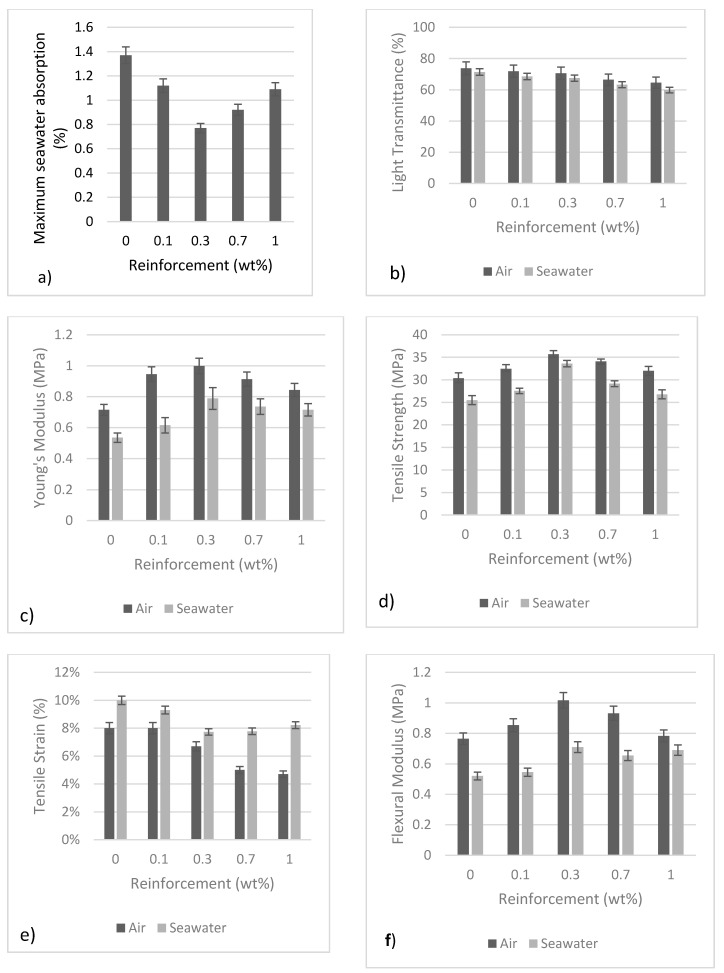

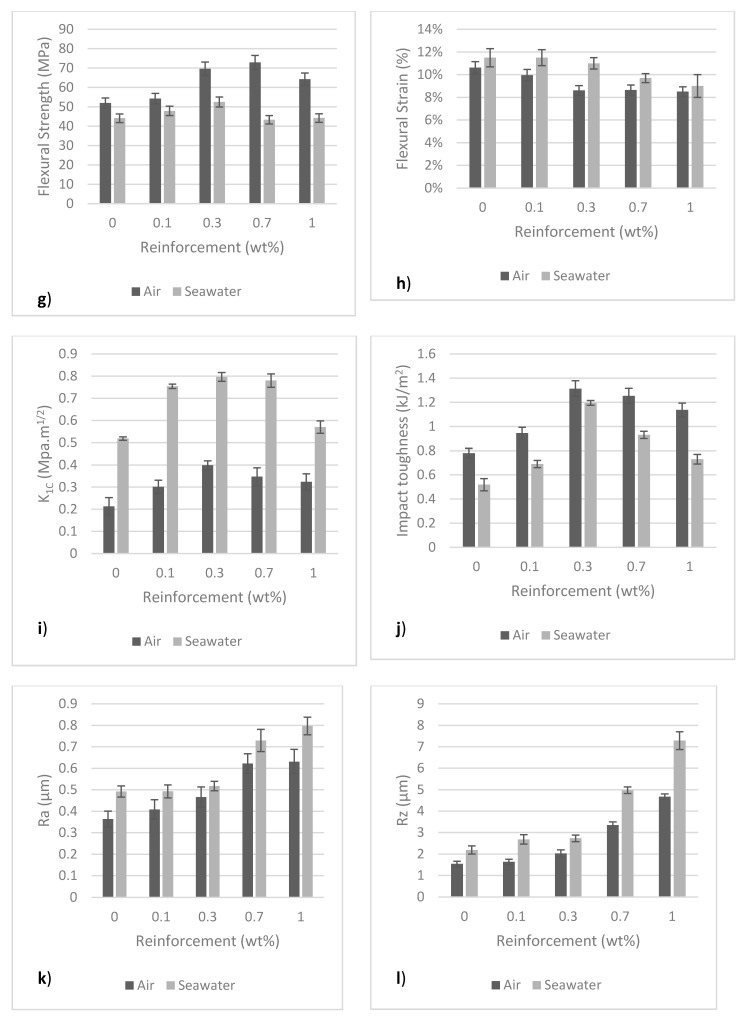
Mechanical properties of nanocomposites; (**a**) seawater absorption; (**b**) light transmission; (**c**) Young’s modulus; (**d**) tensile strength; (**e**) tensile strain (**f**) flexural modulus; (**g**) flexural strength; (**h**) flexural strain; (**i**) fracture toughness; (**j**) impact toughness; (**k**) Ra; and (**l**) Rz.

The maximum seawater absorption is presented in Figure 5a. After five days of seawater exposure, monolithic polyester recorded the highest seawater absorption with 1.37%. The seawater absorption reduced with 0.1 wt % HNTs reinforcement. The 0.1 wt % HNTs-polyester recorded 1.12% seawater absorption. At 0.3 wt % HNTs reinforcement, the seawater absorption recorded the minimum value. Only 0.77% seawater absorption was observed in this nanocomposites system, which is 0.6% lower than monolithic polyester. The seawater absorption result indicates, HNTs act as liquid barrier for the nanocomposites [43]. Some scholars suggested the incorporation of HNTs twisted the liquid path length and reduce the liquid penetration [44,45]. In contrast, monolithic polyester does not have similar path length. The liquid tends to penetrate from surface through microvoids which further damage the polymer matrix. The variation of the light transmittance in air and after seawater immersion is presented in Figure 4b.

The light transmittance was performed between 300 and 1400 nm and the wavelength was selected at 400 nm based on previous literature by Bharadwaj et al. [46]. The light transmittance graph can be used to justify the quality of dispersion but also the effect of seawater on optical clarity. Regarding dispersion of the nanocomposites, the light transmittance decreases with the increase of HNTs concentration. After sonication, the aggregates are evenly distributed into small particles causing the increase in light absorption or reduce light transmittance [47]. The light transmittance values were higher for unexposed samples, but the opposite trend was observed for samples exposed in seawater. For instance, monolithic polyester has the highest light transmittance. However, after seawater immersion the light transmittance dropped from 73.8% to 71.4%. This indicates a slight deterioration in the optical clarity of the monolithic polyester. For the other nanocomposites used in this study, similar trends were also onserved.

Figure 5c shows the Young’s modulus of the nanocomposites. Monolithic polyester showed the lowest value with 0.71 MPa. The modulus increased at 0.1 wt % reinforcement with 0.95 MPa. The maximum increase in Young’s modulus was observed in nanocomposites reinforced with 0.3 wt % HNTs. The Young’s modulus increased to 41% compared to monolithic polyester. For samples tested in air, the Young’s modulus was significantly enhanced with the addition of 0.1, 0.3, 0.7 and 1 wt % HNTs. In contrast, after seawater exposure, all nanocomposites were having slightly lower Young’s modulus as evident in Figure 5c. However, HNTs were found to have remarkable influence on the polyester matrix. For instance, at 0.3 wt % reinforcement, the Young’s modulus dropped 20% while monolithic polyester dropped up to 24%.

The variation of tensile strength is presented in Figure 5d. The maximum tensile strength was recorded in the case of 0.3 wt % reinforcement. The tensile strength increased from 30.4 to 36 MPa (i.e., an improvement of 18%). The exfoliated HNTs reduced the mobility of polymer chains during tensile loading [41]. The presence of HNTs also slowed or pinned the crack growth, which resulted in an improvement of tensile modulus. After seawater immersion, the tensile strength was found to be reduced for all the nanocomposites. The average tensile strength of monolithic polyester, reduced from 30.4 to 25.5 MPa. The seawater caused 16% of tensile strength reduction. For 0.3 wt % reinforcement, the tensile strength reduced from 36 to 34 MPa, which indicate only 6% of tensile strength reduction.

The tensile strain is also presented in Figure 5e. In general, the tensile strain decreased with the increase of HNTs for samples tested in air. Monolithic polyester and 0.1 wt % HNTs reinforcement recorded higher tensile strain of 8% while 0.3, 0.7 and 1 wt % HNTs-polyester were having the tensile strain between 5% and 7%. After seawater exposure, the tensile strain increased as the stiffness of the nanocomposites declined. The tensile strain also suggests degradation behavior of the samples after seawater exposure. The retardation of degradation in polymers exposed to the elements while exposed in seawater is primarily the result of the relatively lower temperatures and the lower oxygen concentration in water environments [27].

Figure 5f illustrates the flexural modulus of the nanocomposites before and after seawater exposure. The maximum flexural modulus was obtained at 0.3 wt % reinforcement where 23% of improvement was observed for samples tested in air. The data show similar trend as presented in Young’s modulus. After seawater exposure, 29% of flexural modulus reduction was observed, however the result is still better than monolithic polyester. Even though the flexural modulus reduction is unavoidable, the presence of HNTs enhanced the stiffness of the polyester matrix.

Compared to monolithic polyester, the presence of HNTs improved the flexural modulus of the nanocomposites. The variation of flexural strength is presented in Figure 5g. The maximum flexural strength recorded was from 52 to 70 MPa in the case of 0.3 wt % reinforcement. At higher HNTs reinforcement, the flexural strength of unexposed samples was slightly decreased. The average values for 0.7 and 1 wt % reinforcement was 68.2 and 64.2 MPa respectively. The lower values can be linked to the morphological structure of these nanocomposites, the presence of intercalated aggregates that was poorly dispersed. These aggregates created stress concentrations in the polyester thus reduced the flexural strength. After seawater exposure, the flexural strength decreased because of several factors. The degradation of the samples is due to the synergistic effect of the polymer matrix softening and microbial attack. The failure mode was changed from brittle to ductile failure The moisture from seawater caused biodegradation and chemical deterioration [48,49]. The flexural strains are illustrated in Figure 5h. The main difference between samples tested in air and seawater can be seen in flexural strain. Samples tested in air tend to have shorter flexural strain while samples exposed in seawater have slightly higher values. As described earlier, this is typical phenomenon for degraded samples [42].

The variation of fracture toughness is presented in Figure 5i. In normal environment (i.e., open air conditions), the maximum fracture toughness was observed for 0.3 wt % HNTs reinforcement. The fracture toughness improved from 0.21 to 0.4 MPa·m^1/2^ (90% increase). The exfoliated structure increased toughness properties towards the loading directions due to the high aspect ratio. Increases in fracture toughness are mainly due to crack bridging, crack deflection and plastic deformation of the polyester around the halloysite particles [50]. Halloysite nanotubes can interact with cracks at the crack front, resisting the advance of the crack and thus improving the fracture toughness [51].

After seawater exposure, the fracture toughness increased for all nanocomposites due to plasticization effect of the polymer matrix on the surface layer [52]. Sobrinho et al. also revealed the plasticization effect produced endothermic reactions by water sorption and crosslink breaking [3]. Other researchers also reported an increase in fracture toughness for nanocomposites exposed to moist environment [12,21,53]. The increase of ductility due to the absorbed moisture tended to increase fracture toughness. The impact toughness is presented in Figure 5j. At 0.3 wt % reinforcement, HNTs have significantly increased the impact toughness from 0.78 to 1.3 kJ/m^2^ (increase 67%). At 0.7% reinforcement, the impact toughness improved about 60% from 0.78 to 1.25 kJ/m^2^. At 1 wt % HNTs, the impact toughness improved 46%.

For samples tested in air, it can be seen that HNTs remarkably increased their impact toughness values. After seawater immersion, the impact toughness decreased compared to dry samples. Monolithic polyester recorded the minimum value, which is 0.52 kJ/m^2^. The maximum impact toughness was recorded in the case of 0.3 wt % HNTs-polyester. The impact toughness increased from 0.52 to 0.9 kJ/m^2^. The average surface roughness R_a_ is shown in Figure 5k. In general, samples reinforced with higher HNTs concentrations show higher average surface roughness value. Monolithic polyester recorded the minimum average surface roughness with 0.36 µm. The surface roughness increased steadily with the increase of HNTs. At 0.1 and 0.3 wt % HNTs-polyester, the surface roughness values were 0.4 and 0.47 µm, respectively. At higher HNTs (1 wt % HNTs-polyester), the surface roughness increased up to 0.63 µm (increase 73%). The surface roughness for all nanocomposites slightly increased after seawater exposure. This is due to the plasticized polymer matrix and penetration of bacteria which produced coarser topography [54]. Penetration of bacteria which lead to an increase of coarser topography also known as surface extension of the material as described by Scaffaro et al. [55]. Surface cavities and unevenness also contributing to the coarser topography [55,56]. The R_z_ (highest and lowest point of profile) showed in Figure 5l also shows similar trend as R_a_, which indicates coarser profile after seawater exposure.

### SEM Images

**Figure 6:**
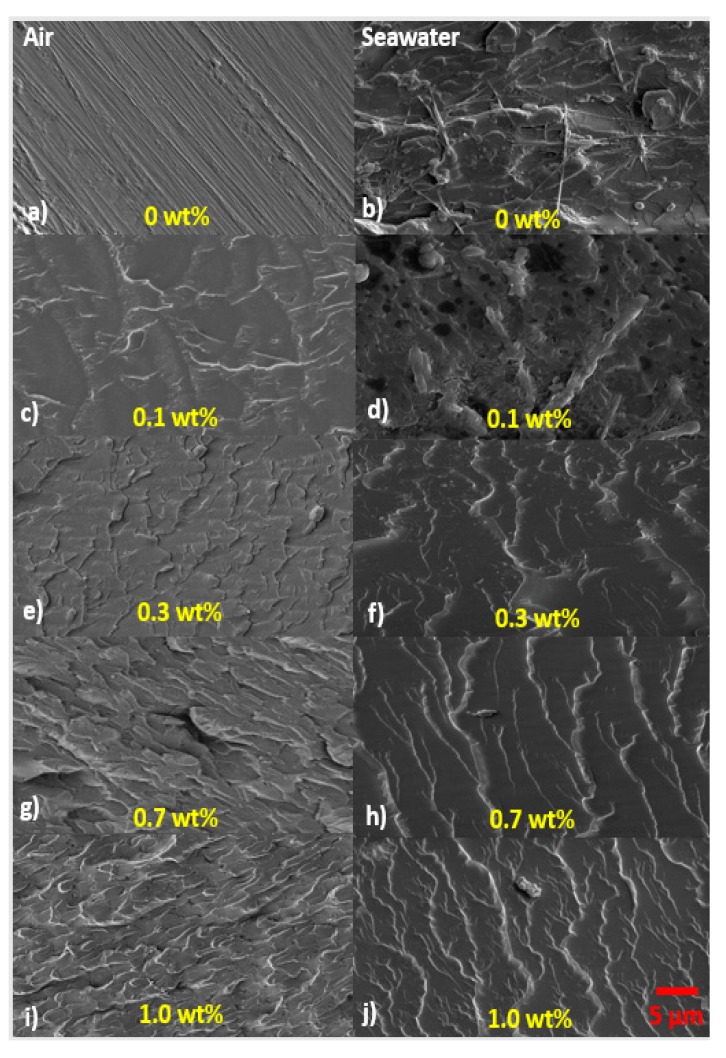
SEM micrographs of nanocomposites in air and after seawater exposure (scale at the bottom).

**Figure 7:**
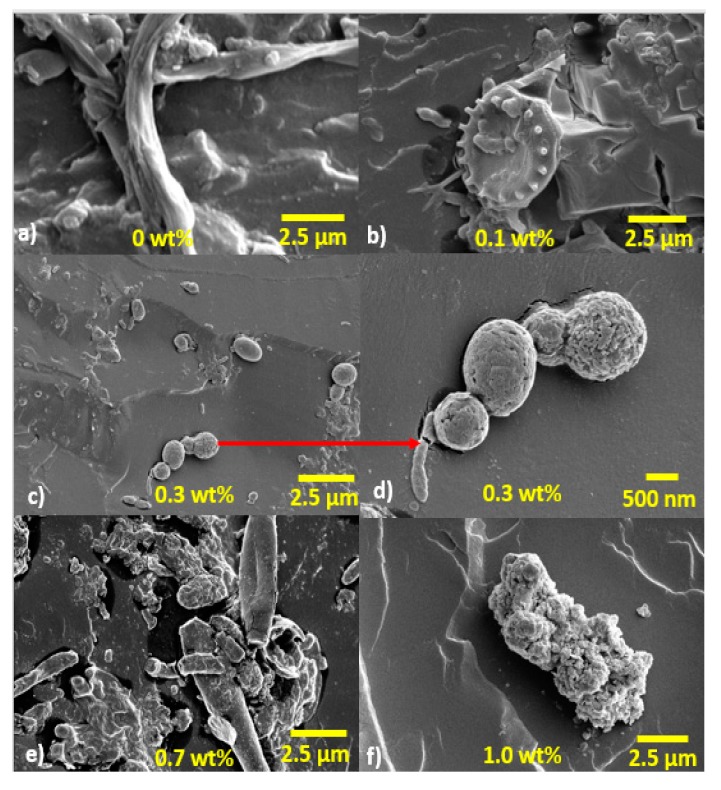
SEM micrographs of microbes on nanocomposites after seawater exposure.

The influence of HNTs was viewed using Scanning Electron Microscope on fractured surfaces of specimens. The monolithic polyester in dry condition is presented in Figure 6a. The image shows almost featureless surface because of straight and direct crack propagation [57]. The evidence of exfoliated morphology for nanocomposites is presented in Figure 6c,e,g,i. Exfoliated morphology increase surface roughness and produce the highest surface area interaction between halloysite and the matrix [58]. When exfoliation has been achieved, the mechanical properties and barrier properties are significantly enhanced [33,58]. The increase in surface roughness can be linked to crack deflection mechanism produced from HNTs addition. The improvement in surface roughness means that the fracture path also increases, as a result, the stiffness and the strength of the nanocomposites were significantly enhanced. The cracks also radially emanated as lower as 0.1 wt % HNTs reinforcement. The SEM images of fractured surface for monolithic polyester and its nanocomposites after seawater immersion are presented in Figure 6b,d,f,h,j. In general, seawater exposure significantly altered the fracture path from radial to straighter crack propagation [39]. The most affected specimens were monolithic polyester and 0.1 wt % HNTs-polyester samples, where the presence of microbes can be observed at low magnification. For 0.3, 0.7 and 1 wt % samples, the addition of HNTs was responsible to reduce the effect of plasticization and seawater penetration. At higher magnification, different size microbes can be spotted in all nanocomposites systems. The bacteria size observed in the SEM images are within the range between 0.2 and 5 µm as reported in literature [59]. Filamentous cyanobacteria, as shown in Figure 7a, covered most of the monolithic polyester fractured surface layers. This microorganism, can create a microenvironment where polyester matrix become chemically unstable [28]. Filamentous cyanobacteria with diameter between 500 and 550 nm are likely to attack monolithic polyester. This is possibly due to presence of larger voids during processing. In contrast, HNTs fill the voids and reduce the chance of filamentous cyanobacteria entry. As for the nanocomposites reinforced with 0.1 wt % HNTs-polyester, fungal and several marine bacteria such as Centropyxis were observed, as shown in Figure 7b. Staphylococcus type bacteria on the other hand were found attached to the 0.3 wt % HTS-polyester nanocomposites, as presented in Figure 7d. Another bacteria known as *Aeruginosa* is presented in Figure 7e,f found in 0.7 and 1 wt % HNTs-polyester nanocomposites. The microscopic images show the diversity in the shape and sizes of bacteria that observed on the nanocomposites. All marine bacteria regardless of their shapes, are responsible for actively breaking off hydrocarbon polymers using water molecules. Bacteria are not visible with naked eye because of their small size. The size of bacteria cell is between 0.2 and 5 μm. The image presented here is identical to what has been reported in the literature [60,61]. Detached bacteria from polymer matrix can create pores size between 3 and 5 μm. Cavities and unevenness created from detached bacteria would allow seawater penetration even faster. Changes in visual effect, such as yellowing of the polyester caused by biofilm formation on the surface of the samples, was also observed. In addition, various microbial communities that had developed on the nanocomposites may release acid compounds such as Nitrosomonas. The Chemoorganotrophic communities released the organic compound with acidic features, such as oxalic, gluconic, fumaric, citric and glutaric [59]. These acid,s capable of changing the pH within the microvoids, consequently create so-called gradual biodegradation on the microstructure of the polyester matrix. The deterioration at the interface is caused by hydrolysis, which is caused by longer immersion time and sustained seawater led to disintegration of the chemical bonds at the interface resulting in separation of halloysite and polyester matrix. Hydrolysis causes swelling in the matrix, which leads to decrease in mechanical adhesion between the halloysite and polyester matrix. Along with plasticization effect, the mechanical properties were reduced as the ductility of the polyester matrix increased. Some studies reported that seawater absorption causes plasticization and hydrolysis effect [49]. The failure mode also involved alteration from brittle matrix to ductile, as a consequence of seawater diffusion.

## 4. Conclusions

Monolithic polyester and its nanocomposites of four different concentrations of HNTs reinforcement were successfully produced and the biodegradation of their mechanical properties was studied after 120 h. HNTs are capable of increasing storage modulus and glass transition temperature (*T*_g_) by increasing the stiffness of nanocomposites and restricting polymer chains. In this research, the addition of HNTs strengthened the polyester matrix up to a concentration of 0.3 wt %. The tensile properties and flexural properties were remarkably improved compared to monolithic polyester. Seawater exposure significantly reduced the storage modulus and glass transition temperature of all samples. The tensile properties and flexural properties were also decreased. In contrast, fracture toughness and surface roughness increased due to factors such as plasticization effect and the presences of microbes on the samples fractured surface. Microbes can cause chemical degradation and breaking of hydrocarbon using seawater molecules. Nanocomposites biodegradation is highly undesirable to material integrity, as these are used mostly in structural designs of marine applications. Damage to the structure may result in premature weakening, which is often translated to system failure and enormous economic losses.

## Figures and Tables

**Table 1:** Mechanical properties of clay based composites from literatures.

Nr	Authors	Year	Reinforcement/(wt %)	Polymer	Mechanical Properties	Max Increase (%)	Ref.
1	Albdiry et al.	2013	Halloysite nanotubes/3	Unsaturated polyester	Impact strength	16	[29]
2	Lin et al.	2011	Halloysite nanotubes/5	Epoxy	Impact strength	300	[30]
3	Chozhan et al.	2008	Clay/3	Epoxy	Impact strength	19.2	[31]
4	Ye et al.	2007	Halloysite nanotubes/2.3	Epoxy	Impact strength	413	[32]
5	Sancaktar	2011	Nanoclay/1	Epoxy	Young’s modulus	11	[33]
6	Carli et al.	2011	Halloysite nanotubes/5	PHBV	Young’s modulus	63	[22]
7	Liu et al.	2001	Nanoclay/5	Epoxy	Young’s modulus	40	[34]
8	Lepoittevin	2002	MMT/10	PCL(Poly(ε-caprolactone)	Young’s modulus	54	[35]
9	Alamri and Low	2012	Halloysite/5	Epoxy	Flexural Modulus	88	[36]
10	Pavlidou et al.	2008	MMT/5	Epoxy	Flexural modulus	224	[23]
11	Manfredi et al.	2008	Cloisite/5	Epoxy	Flexural modulus	29	[37]
12	Wetzel et al.	2006	MMT/10	Epoxy	Flexural modulus	40	[38]
